# Incidence of Maxillary Sinus Disorders in Dental Patients Undergoing Cone-Beam Computed Tomography: A Retrospective Cross-Sectional Study

**DOI:** 10.7759/cureus.71114

**Published:** 2024-10-08

**Authors:** Sawsan Saidahmed, Sarra Ali, Meaad Elbashir, Bashair Abu Habiba, Hana Mohammed, Belqees Hakami, Sarra Kamal, Asma Alamin, Esam S Alshameri, Ali Abdelrazig, Awadia Gareeballah

**Affiliations:** 1 Diagnostic Radiography Technology, College of Nursing and Health Sciences, Jazan University, Jazan, SAU; 2 Medical Laboratory, College of Nursing and Health Sciences, Jazan University, Jazan, SAU; 3 Diagnostic Radiology, College of Applied Medical Sciences, Hail University, Hail, SAU; 4 Maxillofacial Surgery, Pure Smile Dental Center, Jazan, SAU; 5 Diagnostic Radiology, College of Applied Medical Sciences, Taibah University, Madinah, SAU; 6 Diagnostic Radiologic Technology, Faculty of Radiology Science and Medical Imaging, Alzaiem Alazhari University, Khartoum, SDN

**Keywords:** cbct, incidental findings, maxillary sinuses, opacification, orthodontics

## Abstract

Background

Cone-beam computed tomography (CBCT) was quickly adopted in dentistry settings due to its compact size, low cost, and minimal ionizing radiation dose when compared to medical computed tomography. CBCT generates three-dimensional (3D) images that are useful in a variety of oral and maxillofacial circumstances, including diagnosis and assessment of disease severity, treatment planning and delivery, and follow-up. A significant advantage of CBCT lies in its capability to recognize incidental findings. In the realm of dental CBCT imaging, incidental findings often involve the maxillary sinuses, temporomandibular joints, or other craniofacial structures.

Objective

To demonstrate the incidental findings of maxillary sinus disorders in dental patients undergoing CBCT for numerous indications.

Methods

A retrospective cross-sectional study was carried out using data extracted from the Picture Archiving and Communication System (PACS) of Pure Smile Dental Center and Dental College Teaching Hospitals in the Jazan region from September to December 2022. The study sample included a total of 70 patients who underwent orthodontics CBCT, most of whom were female (76%). Statistical analysis was performed using IBM SPSS Statistics for Windows, Version 27 (IBM Corp., Armonk, USA), along with descriptive statistics. Furthermore, chi-square tests and cross-tabulation were done to assess the relation between the study variables. A p-value <0.01 and <0.05 was considered statistically significant.

Results

The most common dental CBCT findings were root invasion (18.6%), followed by gingivitis (7.14%), and dental caries (4.29%). Of the 70 patients, 42 (60%) had maxillary sinus findings, including opacification (50%), sinusitis (40%), mucosal thickening (34.3%), and polyps (7.1%). Female patients had more frequency of incidental findings related to males (41.43% and 18.57%, respectively).

Conclusion

A high percentage of dental patients exhibited incidental maxillary sinus findings in CBCT scans, confirming the utility of CBCT as a precise imaging method for detecting paranasal sinus disorders. A holistic documentation of incidental findings is crucial for improving patient outcomes and treatment plans.

## Introduction

Cone-beam computed tomography (CBCT) has emerged as a valuable imaging acquisition in orthodontics, offering detailed three-dimensional (3D) imaging of dental and maxillofacial structures. This technology provides critical information for treatment planning, including tooth position, bone structure, and airway analysis, while minimizing radiation exposure compared to traditional computed tomography (CT) scans [1,2]. The development of specialized CBCT scanners for use in dentistry began in the latter part of the 1990s. Soon after, the usage of CBCT for dental, maxillofacial, and ear-nose-throat applications increased dramatically. CBCT is now widely utilized for a variety of dental applications, including implant design, endodontics, maxillofacial surgery, and orthodontics [2]. Furthermore, CBCT’s expanded field of view (FOV) often reveals incidental findings in adjacent anatomical areas, such as the paranasal sinus, which are beyond the primary scope of orthodontic evaluation [3,4].

The maxillary sinus, due to its proximity to the upper dentition and maxillofacial region, is frequently affected. Incidental findings such as mucosal thickening, sinusitis, and polyps are commonly observed in CBCT scans. Recent literature provides substantial evidence that dental sepsis frequently leads to reactive mucosal thickening in the lower part of the maxillary sinus. It is now recognized that odontogenic factors are a more common cause of maxillary sinusitis, including sinusitis affecting the anterior paranasal sinus, than was previously believed, with incidence rates on the rise. In fact, dental issues account for 75% of opacification in the maxillary sinus and 25-40% of cases involving unilateral anterior sinusitis [5,6]. These findings, while often asymptomatic, may have clinical significance, potentially influencing orthodontic treatment decisions or requiring referral for further medical evaluation. Studies suggest that a substantial proportion of CBCT scans reveal incidental findings in the maxillary sinus, commonly mucosal thickening, highlighting the importance of routine evaluation of these structures during orthodontic assessment [7,8].

Despite the growing use of CBCT in orthodontic practice in Saudi Arabia, particularly in the Jazan region, there is limited data on the prevalence and nature of incidental maxillary sinus findings in this population. Understanding these incidental findings is crucial for promoting interdisciplinary collaboration between orthodontists and other specialists, such as otolaryngologists, for comprehensive patient care. Therefore, the main aim of this study was to determine the prevalence of incidental maxillary sinus findings in CBCT scans among orthodontic patients in the Jazan region.

## Materials and methods

Study design and data collection

A cross-sectional retrospective study was conducted at Pure Smile Dental Center and Dental College Teaching Hospitals in the Jazan region between September and December 2022. The study protocol received verbal approval from the ethical committee of the Diagnostic Radiography Technology Department at Jazan University. A total of 70 orthodontic patients, 53 females and 17 males, indicated for CBCT dental scans were included. Patients with known sinus disease and children under 12 years old were excluded. Data were collected using a data collection sheet, including the participant demographic information, dental scan indications, CBCT dental findings, and incidental maxillary sinus disorders.

Equipment and technique

CBCT was performed with a 3D X-ray imaging system (3D Accuitomo 170; J. Morita USA, Inc.) set at 0.4 mm slice thickness, 16x22 FOV, 20 seconds scan time, and 0.49/0.49/0.5 voxel size, 90 kVp, 7 mA, 15.8 seconds exposure time. The CBCT approach performed on patients in sitting position involves a circular or rectangular cone-shaped X-ray beam with a single 360° scan, wherein the X-ray source and a reciprocating array of detectors revolve around the patient's head stabilized by a head holder. Single projection images, known as "basis" images, are obtained at certain degree intervals similar to lateral cephalometric radiography images but slightly offset from one another. The projection data is a collection of such basis projection images used by software programs with sophisticated algorithms to generate a 3D volumetric data set that can be used to provide primary reconstruction images in all three orthogonal planes (axial, sagittal, and coronal). Multiple imaging reconstructions and measurements according to the indications were performed. In CBCT, the smaller FOV is used for periapical evaluation of selected teeth, alveolar bone, and a limited area of maxillary or mandibular bone. On the other hand, larger FOVs include the cervical spine, jaws, paranasal sinuses, skull base, and parts of the cranium. In this study, the 3D CBCT using a larger FOV was diagnosed by an experienced radiologist to assess the incidental findings in maxillary and facial structures.

Data analysis

The data collected in this study was analyzed using IBM SPSS Statistics for Windows, Version 27 (IBM Corp., Armonk, USA) and DATAtab Online Statistics Calculator (DATAtab e.U. Graz, Austria). Descriptive statistics were obtained using frequencies and percentages. Cross-tabulation using chi-square tests was employed to assess the relation between demographic data and incidental CBCT findings. A p-value <0.01 and <0.05 was considered statistically significant.

## Results

The results demonstrate that the mean age of orthodontics patients who presented to conduct a CBCT scan was 33.66±13.44 years. Most patients (52.9%) were aged between 15 and 30 years, followed by 28.6% aged between 31 and 46 years. More than two-thirds of the participants in this study were female (75.7%) (Table 1). 

**Table 1:** Frequency distribution of age and gender The frequency of age group and gender among 70 orthodontic patients who underwent CBCT. CBCT: Cone-beam computed tomography

Demographic characters		Frequency	Percent
Age groups	15-30	37	52.9
	31-46	20	28.6
	47-62	11	15.7
	63-78	2	2.9
Gender	Female	53	75.7
	Male	17	24.3
	Total	70	100.0
Mean age ± SD (33.66±13.44 years)			

Concerning the dental findings, the study revealed that most of the patients suffered from incidental maxillary sinus disorders (60%) (Table 2).

**Table 2:** Frequency distribution of detected incidental maxillary sinus findings Percentage of incidental findings in maxillary sinuses (60%).

Incidental maxillary sinus disorders	Frequency	Percent
No	28	40.0
Yes	42	60.0
Total	70	100.0

**Figure 1:**
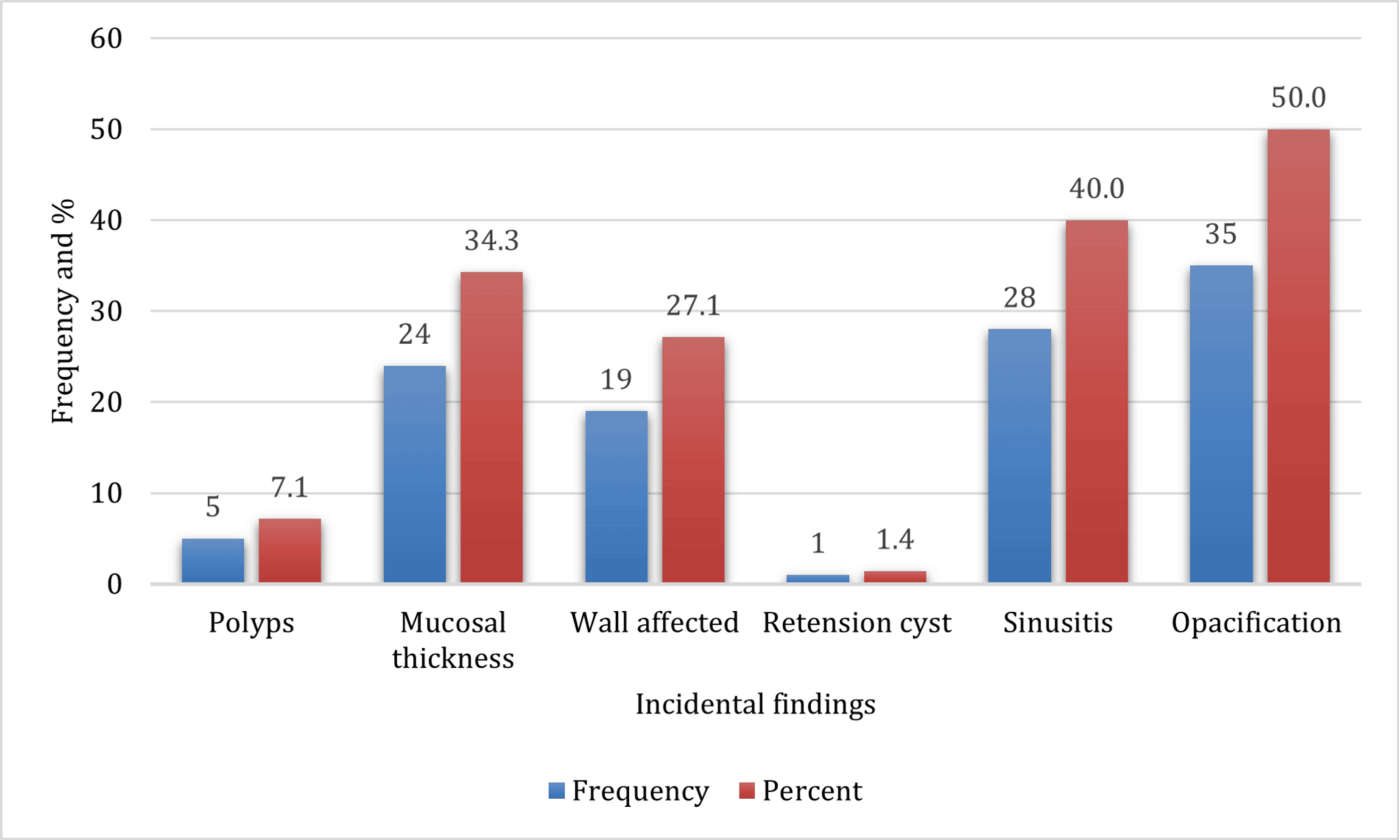
Frequency distribution of incidental maxillary sinus findings Incidental maxillary sinus disorders in CBCT. CBCT: Cone-beam computed tomography

**Figure 2:**
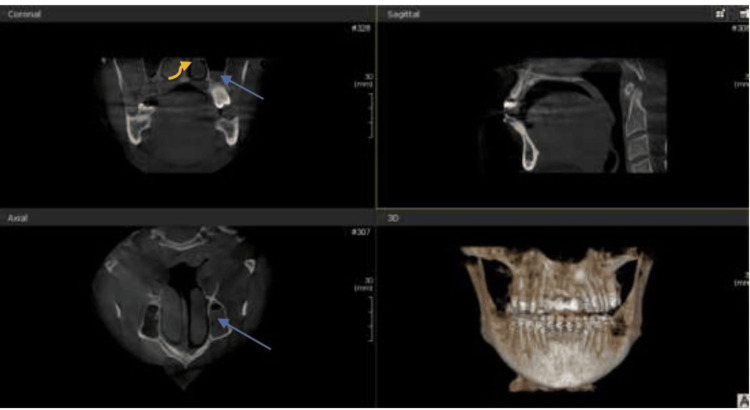
Left maxillary sinus opacification (blue arrows in both coronal and axial section) and deviated nasal septum (yellow curved arrow in coronal section) in a patient who presented for nerve treatment Coronal, axial, and sagittal CBCT images showing incidental maxillary and nasal findings in an orthodontic patient. CBCT: Cone-beam computed tomography

**Figure 3:**
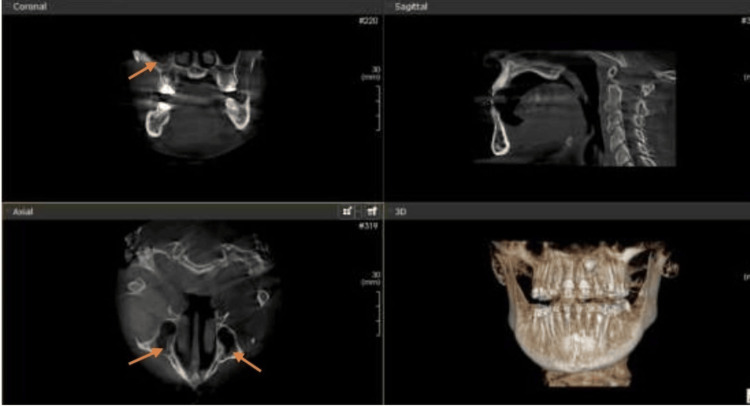
Maxillary sinusitis (orange arrows in both coronal and axial sections) Maxillary sinusitis in coronal and axial CBCT sections of an orthodontic patient. CBCT: Cone-beam computed tomography

**Figure 4:**
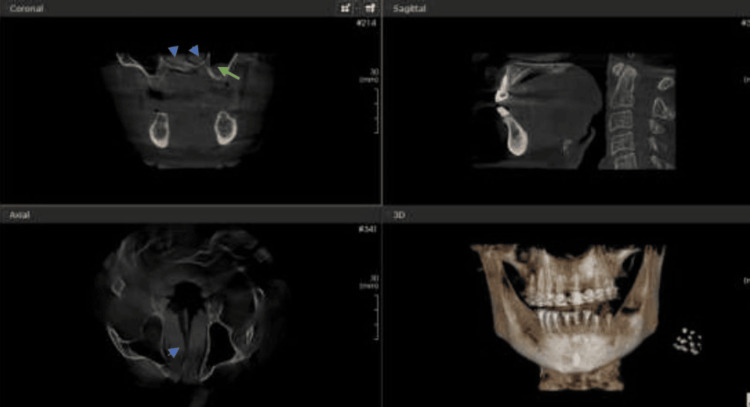
Mucosal thickening (green arrow in coronal section) and hypertrophy of inferior nasal turbinate (blue arrow heads in both coronal and axial sections)

Among the incidental maxillary sinus findings detected, opacification was the most prevalent, occurring in 50% of the cases, followed by sinusitis in 40% and mucosal thickness in 34.3% of the cases. Wall-affected findings were noted in 19% of the cases. The least common associated incidental findings were retention cysts and polyps (Figures 1-4).

**Figure 5:**
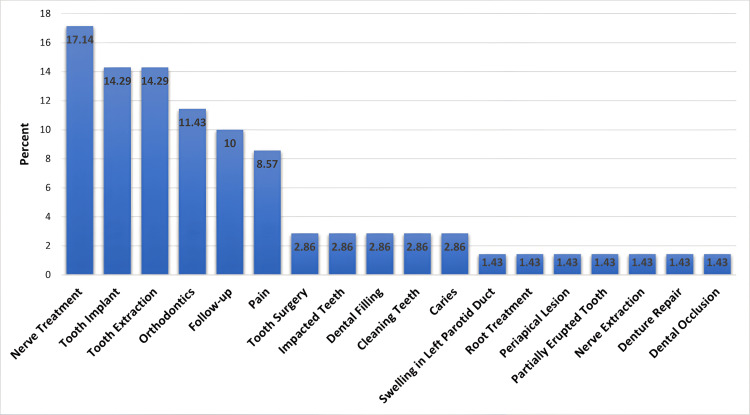
Frequency distribution of dental indications

The most common dental indications were nerve treatment (17.14%), tooth extraction (14.29%), tooth implant (14.29%), and follow-up (10%) (Figure 5).

Several dental findings were noted, with root invasion being the most common at 18.57%. Other findings included gingivitis (7.14%) and the less frequent ones such as dental caries (4.3%), malocclusion, and irreversible pulpitis. Conditions like impacted teeth, periapical lesions, and periodontitis were noted with lower frequencies (Table 3).

**Table 3:** Dental findings on CBCT CBCT: Cone-beam computed tomography

Dental findings	Percent
Normal	55.72%
Roots invasion	18.57%
Gingivitis	7.14%
Dental caries	4.29%
Impacted tooth	2.86%
Reversible pulpitis	1.43%
Pneumatization	1.43%
Periodontitis	1.43%
Periapical lesion around palatal	1.43%
Palatal impacted	1.43%
Malocclusion	1.43%
Irreversible pulpitis	1.43%
Total	100%

In Table 4, when analyzing the types of findings across different age groups, mucosal thickening was more common in the 31-46 and 47-62 age groups, with a total of 24 cases observed. Significant differences were noted among different age groups (p=0.031). Other findings such as polyps, wall defects, retention cysts, and opacifications did not show significant age-related variations. About 35 cases of opacifications were evenly distributed across all age groups, with most cases discovered in age groups 15-30 and 31-46. Sinusitis was more prevalent in age groups 31-46, with no statistically significant differences across different age groups (p=0.327).

**Table 4:** Cross-tabulation findings and age group

Finding		Age group				Total	p-value
		15-30	31-46	47-62	63-78		
Mucosal thickening	Yes	8	9	7	0	24	0.031
	No	29	11	4	2	46	
Polyp	Yes	4	0	1	0	5	0.474
	No	33	20	10	2	65	
Wall affected	Yes	7	7	5	0	19	0.212
	No	30	13	6	2	51	
Retention cyst	Yes	1	0	0	0	1	0.824
	No	36	20	11	2	69	
Opacifications	Yes	18	11	5	1	35	0.957
	No	19	9	6	1	35	
Sinusitis	Yes	1	16	5	6	28	0.327
	No	1	21	15	5	42	

**Figure 6:**
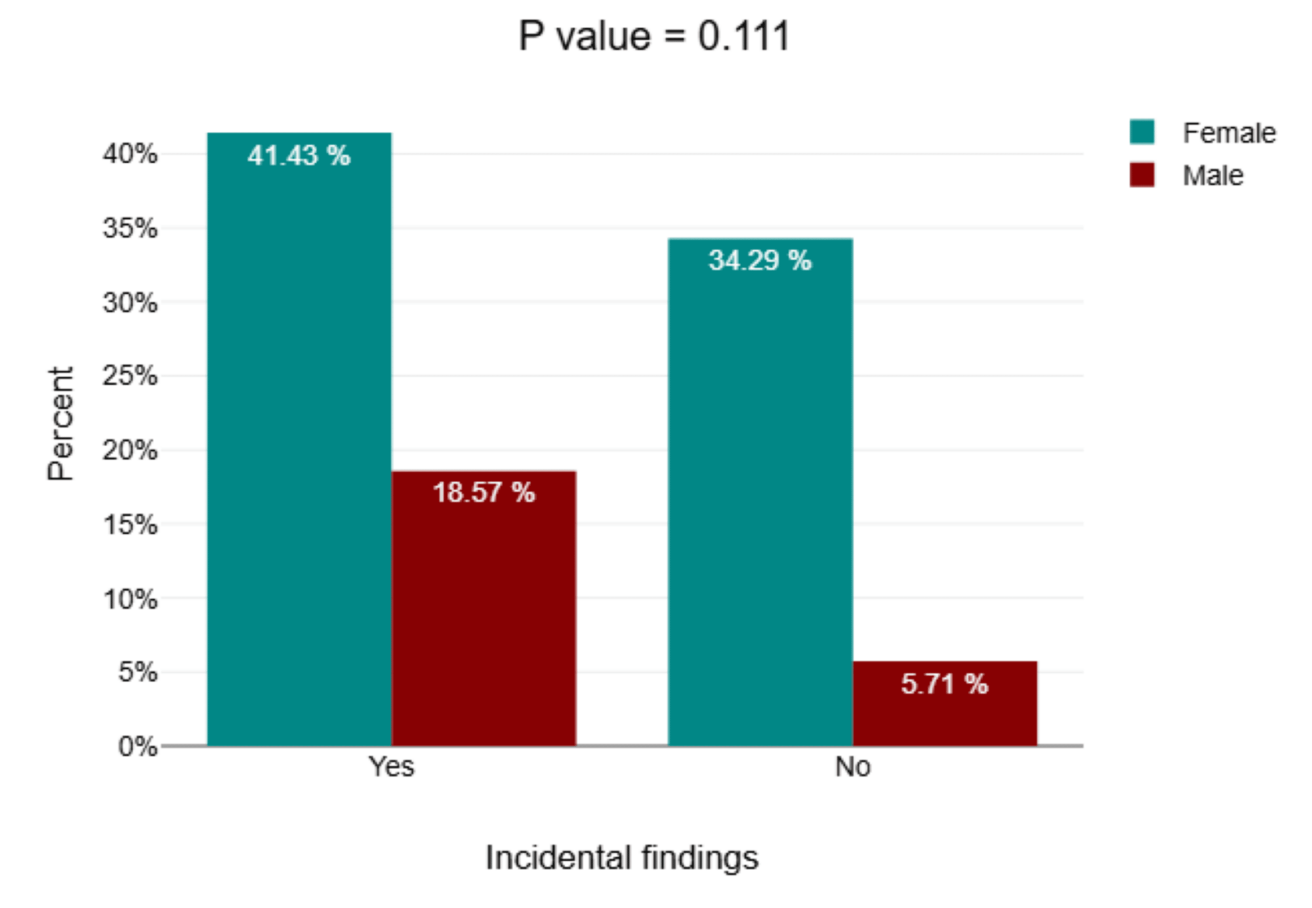
Cross-tabulation gender and incidental findings Bar chart showing females mostly affected by incidental maxillary findings.

The bar chart demonstrates the distribution of incidental findings in CBCT scans among both genders. It shows that incidental findings were more commonly detected in females, with 41.43% of the total cases (29/70) compared to 18.57% (13/70) in males. Contrariwise, the absence of incidental findings was noted in 34.29% of females (24/70) and only 5.71% of males (4/70). The difference in the incidence of incidental findings between males and females was not statistically significant (p=0.111) (Figure 6).

**Figure 7:**
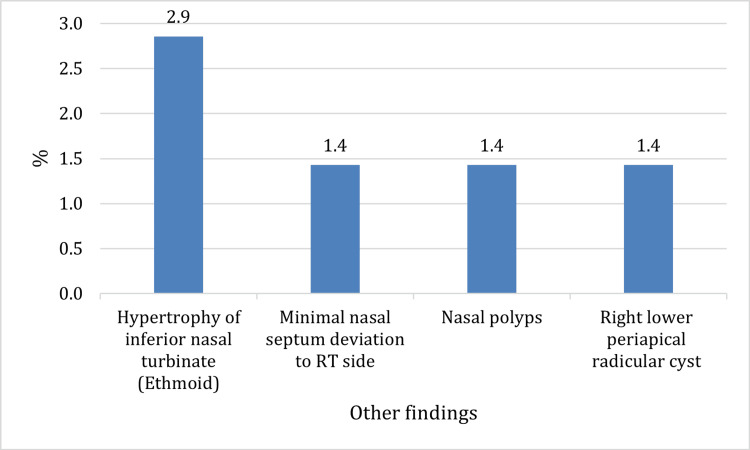
Other incidental findings Bar chart showing other incidental findings. RT: Right

Other incidental findings were noted in the nose and periapical areas such as hypertrophy of the inferior nasal turbinate (ethmoid) with a prevalence of 2.9%, nasal polyps, and right lower periapical radicular cyst (Figure 7).

## Discussion

CBCT has been developed in the last few years for applications in dentistry and maxillofacial fields. It is increasingly becoming the preferred imaging technique in particular clinical situations. CBCT offers detailed 3D images of the structures being assessed. CBCT imaging has overcome the limitations associated with two-dimensional (2D) imaging, providing practitioners with high-quality images that possess sub-millimeter resolution, while also ensuring a short scanning duration and reduced radiation exposure. As this technology becomes an integral part of dental practice, reliance on practice-based estimations will diminish, resulting in advantages for both patients and dentists [9]. The maxillary sinuses are the most substantial paranasal sinuses to assess in dentistry because of their closeness to the upper teeth. Consequently, conditions affecting the maxillary antra may mimic odontogenic infections, and issues related to the teeth can also affect the maxillary antra or exhibit symptoms similar to those associated with antral diseases [10]. In this study, the common reasons for CBCT were nerve treatment, tooth implants, and tooth extractions. In contrast, Ritter et al. noted that trauma and dental implants were the primary reasons for CBCT [7]. Meanwhile, Younis et al. reported that the most frequent reason for CBCT was related to tooth implant planning [10].

This study offers a comprehensive analysis of CBCT scans in orthodontic patients, revealing critical insights into incidental findings and dental indications. The mean age of the patients undergoing CBCT scans was 33.66±13.44 years, with the majority (52.9%) aged between 15 and 30 years. Moreover, over two-thirds of the participants were female. Consistent with this study, Lagorsse et al. in a literature review mentioned a higher prevalence of young female patients in orthodontic settings, probably because of their higher aesthetic demands [11].

The incidental maxillary findings detected in a majority of orthodontic patients (60%) underscores the importance of evaluation of CBCT images to assess the associated dental and maxillary abnormalities as early detection can lead to timely diagnosis, treatment, and follow-up. Opacification, sinusitis, and mucosal thickening were the most prevalent in patients undergoing CBCT. In comparison to this study, Biel et al. found that the prevalence of incidental findings in pre-implant treatment was 82%, with the most common incidental finding in CBCT being the thickening of maxillary sinus mucosa detected in 32% of the patients. Kurtuldu et al. mentioned that mucosal thickening was a common maxillary sinus pathology [12,13]. Similarly, the thickening of maxillary sinus mucosa (29.8%) was found as an incidental finding in 300 CBCT scans, and maxillary sinus mucosal thickening was detected as an incidental finding in 691 CBCT scans [14]. Gracco et al., in a study of 513 CBCT scans performed for orthodontic treatment patients, found that the most frequent incidentals include dental abnormalities, maxillary sinus disorders in half of the cases with mucosal thickening (40.1%), and pseudocysts (10.1%) [15]. Pazera et al. mentioned that incidental maxillary sinus disorders were detected in CBCT orthodontic patients with flat mucosal thickening (23.7%), polypoid mucosal thickening (19.4%), and signs of acute sinusitis (3.6%) [16]. Drage et al., agreeing with this study, mentioned that the incidental findings were very common, occurring in 66% of patients undergoing CBCT scans for orthodontic purposes [17]. Inconsistent with the present study in that the incidental findings were dental in nature, including retained deciduous roots, wisdom teeth, dental aplasia, and dislocation, the orthodontist should be aware of this finding, which may require further investigation, treatment, and follow-up [17,18].

When the types of findings across different age groups were analyzed, mucosal thickening was significantly more common in older age groups. This finding is supported by the research of Gracco et al. [15] who also noted increased mucosal thickening in older age groups. Other findings such as polyps, wall defects, retention cysts, and opacifications did not show significant age-related variations, which is in line with previous studies [15,19]. Gender differences in incidental findings showed that females had a higher prevalence (41.43%) compared to males (18.57%), although this difference was not statistically significant. This finding is consistent with the research of Raghav et al. who also found that the maxillary sinus disorders were 59.7%, with no significant gender differences in incidental findings in CBCT scans [19]. Another study mentioned that the prevalence of incidental maxillary sinus disorders in CBCT scans was 73%, with mucosal thickening and polyps as the most common findings, around 41.4% and 16%, respectively, which suggest significant differences in these findings in both genders. Furthermore, an insignificant difference was found between age groups and sinus abnormalities for CBCT scans [20]. Another study by Alotaibi, who conducted a retrospective analysis of 201 CBCT scans, was inconsistent with this study and mentioned that a significant difference exists between both genders, showing a greater existence of sinus pathology in males compared to females, while in this study, the most common incidental finding was detected in females [21]. The inconsistency observed between the findings of Alotaibi's study and this study concerning gender may be attributed to the smaller sample size in this study.

This study confirmed that the most common incidental CBCT findings in the maxillary sinuses were opacification, sinusitis, and mucosal thickening. Because of their closeness to the upper jaw, there may be a link between periapical lesions and maxillary sinusitis. Near the bottom of the maxillary sinus are the roots of the molars and premolars in the upper back. These teeth may have an advanced periapical infection that erodes the bone, separating the root tip from the sinus and opening the door for infection to enter the sinus cavity via the tooth's root; dental symptoms and sinusitis may result from this. Sometimes an infection from a periapical lesion spreads to the maxillary sinus, resulting in symptoms similar to sinusitis. On the other hand, it can occasionally be challenging to identify the precise origin of discomfort directed to the upper back teeth due to maxillary sinusitis. Not all periapical lesions result in maxillary sinusitis and not all maxillary sinusitis is caused by tooth infections. To properly identify and manage these disorders, dental and medical practitioners must provide proper diagnosis and treatment [22-24].

Other incidental findings such as hypertrophy of the inferior nasal turbinate with a prevalence of 2.9%, nasal polyps, and right lower periapical radicular cysts were also noted. These findings are in line with the study by Rheem et al. who reported incidental findings in dentoalveolar, nasal, and airway regions on CBCT [25]. Furthermore, in another study conducted on the Iranian population, it was found that the prevalence of incidental findings in CBCT images of oral and maxillofacial patients was 60%. The principal frequency of incidental findings were periapical lesions, followed by mucous thickening of the maxillary sinus, retained root, and impaction of the third molar [26]. Smith et al. reported that 19.4% of their patients had a deviated septum and 50.0% had mucosal thickness, which is indicative of maxillary sinusitis [27]. Another study mentioned that 52.1% of patients are diagnosed with mucosal thickening and 6.3% with a deviated nasal septum [28]. The results of this study are largely consistent with previous studies, thus reinforcing the importance of comprehensive evaluation of CBCT images to identify incidental findings of maxillary sinuses and maxillofacial region and improve patients' management and treatment outcomes.

This study had several limitations. It was only a two-center, retrospective study with a small sample size compared to similar studies conducted in the literature. Despite these limitations, the study findings demonstrate similar findings that highlight the diagnostic utility of CBCT in detecting incidental findings in maxillary sinuses and maxillofacial structure, which require clinicians to remain cautious concerning these incidental findings. Future research should focus on these findings through more, multi-center studies to improve their significance and pertinency across diverse populations, thereby refining patient management and outcomes in orthodontics and related fields.

## Conclusions

This study highlights the prevalence of incidental abnormalities in the maxillary sinus noticed in CBCT scans of orthodontic patients. The most common maxillary sinus abnormalities detected were opacifications, sinusitis, and mucosal thickness. A considerable number of the patients were female, and the incidence of sinus-related abnormalities was predominantly high (60%). Other facial abnormalities were noted rather than maxillary sinus abnormalities, accentuating the need for excluding non-dental findings in dental CBCT. Mucosal thickness varied with age, with a higher prevalence noted among the older population. These findings emphasize the necessity of multidisciplinary collaboration between dentists and otolaryngologists in providing complete treatment to patients. Furthermore, it may be helpful to design guidelines for reporting incidental findings in dental CBCT exams to improve diagnostic precision and patient outcomes.
